# Identification of Novel Genetic Variants in CVID Patients With Autoimmunity, Autoinflammation, or Malignancy

**DOI:** 10.3389/fimmu.2019.03022

**Published:** 2020-01-27

**Authors:** Mette Christiansen, Rasmus Offersen, Jens Magnus Bernth Jensen, Mikkel Steen Petersen, Carsten S. Larsen, Trine H. Mogensen

**Affiliations:** ^1^Department of Clinical Immunology, Aarhus University Hospital, Aarhus, Denmark; ^2^Department of Infectious Diseases, Aarhus University Hospital, Aarhus, Denmark; ^3^Department of Biomedicine, Aarhus University, Aarhus, Denmark; ^4^Department of Clinical Medicine, Aarhus University, Aarhus, Denmark

**Keywords:** common variable immunodeficiency (CVID), whole exome sequencing (WES), autoimmunity, granuloma, malignancy, B-cell phenotype

## Abstract

Common variable immunodeficiency (CVID) is a primary immunodeficiency characterized by recurrent bacterial infections and defined by reduced levels of IgG, IgA, and/or IgM, insufficient response to polysaccharide vaccination, and an abnormal B-cell immunophenotype with a significantly reduced fraction of isotype-switched memory B cells. In addition to this infectious phenotype, at least one third of the patients experience autoimmune, autoinflammatory, granulomatous, and/or malignant complications. The very heterogeneous presentation strongly suggests a collection of different disease entities with somewhat different pathogeneses and most likely diverse genetic etiologies. Major progress has been made during recent years with the advent and introduction of next-generation sequencing, initially for research purposes, but more recently in clinical practice. In the present study, we performed whole exome sequencing on 20 CVID patients with autoimmunity, autoinflammation, and/or malignancy from the Danish CVID cohort with the aim to identify gene variants with a certain, possible, or potential disease-causing role in CVID. Through bioinformatics analyses, we identified variants with possible/probable disease-causing potential in nine of the patients. Of these, three patients had four variants in three different genes classified as likely pathogenic (*NFKB1, TNFAIP3*, and *TTC37*), whereas in six patients, we identified seven variants of possible pathogenic potential classified as variants of unknown significance (*STAT3, IL17F, IRAK4, DDX41, NLRC3, TNFRSF1A*, and *PLCG2*). In the remaining 11 patients, we did not identify possible genetic causes. Genetic findings were correlated to clinical disease presentation, clinical immunological phenotype, and disease complications. We suggest that the variants identified in the present work should lay the ground for future studies to functionally validate their disease-causing potential and to investigate at the mechanistic and molecular level their precise role in CVID pathogenesis. Overall, we believe that the present work contributes important new insights into the genetic basis of CVID and particular in the subset of CVID patients with a complex phenotype involving not only infection, but also autoimmunity, autoinflammation, and malignancy.

## Introduction

Common variable immunodeficiency (CVID) is a primary immunodeficiency characterized by recurrent bacterial infections and defined by reduced levels of IgG, IgA, and/or IgM, insufficient response to polysaccharide vaccination, and an abnormal B-cell immunophenotype with significantly reduced fraction of isotype-switched memory B cells. CVID affects an estimated 1 in 25,000 individuals, with an equal distribution of males and females and a broad age range of diagnosis (1, 2). Infections in CVID patients mostly include recurrent pneumonia, sinusitis, middle ear infections, and invasive bacterial infections, particularly with encapsulated bacteria. In addition to this infectious phenotype, at least one third of patients experience autoimmune, autoinflammatory, granulomatous, and/or malignant complications. More specifically, these include immune-mediated thrombocytopenias, hemolytic anemia, lymphoproliferation, splenomegaly, lymphoma/leukemia, enteropathy, nodular regenerative disease of the liver, bronchiectasis, granulomatous lymphocytic interstitial lung disease (GLILD), and ventricular cancer (1, 3–7). Thus, CVID appears to be a condition of dysregulated immunity rather than merely a lack of immunity. This is illuminated by the many parts of the immune system, beyond B cells and antibody that have been ascribed a role in CVID pathogenesis, including abnormalities in Toll-like receptor (TLR) signaling, and altered fractions and functionality of regulatory T cells, B cells, and innate lymphoid cells (1, 6, 8, 9). Importantly, CVID patients with the non-infectious phenotype have a significantly increased risk of complications, tissue damage, and death (5). Moreover, the heterogeneous presentation combined with a very variable age at onset of disease, strongly suggests a collection of different disease entities with somewhat different pathogeneses and most likely diverse genetic etiologies.

The term “common variable immunodeficiency” was first used in 1971 by a World Health Organization committee to distinguish a heterogeneous group of antibody deficiency syndromes from others with a more specific clinical description and well-defined pattern of inheritance (10). The first set of diagnostic criteria for CVID was published by ESID and PASID in 1999 and were largely based on clinical presentation, immunoglobulin levels, and vaccine responses (11). Further description and immunological understanding of CVID was provided by extensive data on immunophenotyping of CVID patients, allowing for the stratification of the disease according to abnormalities in B-cell subsets in the Freiburg classification and EUROclass classifications (3, 12). Importantly, these immunological studies specifically identified a low fraction of isotype-switched memory B cells in a subgroup of patients with an increased risk of autoimmune, granulomatous, and malignant complications. A new set of criteria was subsequently suggested by Ameratunga et al. with a major focus on clinical presentation (13). Most recently, an international consensus document on CVID encompassing the state of the art definitions, knowledge on pathogenesis, and recommendations has been published (14, 15). This document also suggested further genetic analysis as an important aspect for future studies to possibly divide CVID into “infection-predominant,” “inflammation-predominant,” and “autoimmunity” predominant entities aiming at a more precise knowledge of the association between genetics and clinical presentation, disease severity, and immunophenotype (14).

Previously, we described the demographic, infectious, and non-infectious phenotype of the Danish CVID cohort (2). We identified a total of 179 individuals fulfilling diagnostic criteria and reached a prevalence of 1:26,000 among adults in Denmark, with a non-infectious phenotype including autoimmune, autoinflammatory, and granulomatous disease manifestations in approximately one third. In a subgroup of the Danish CVID cohort, a more extensive immunological analysis of lymphocyte populations by multicolor flow cytometry was performed, revealing correlations between altered fractions of regulatory T- and B-cell subsets and autoimmune disease presentation (8). More specifically, we demonstrated an association between elevated levels of regulatory B cells and decreased levels of regulatory T cells and autoimmune phenomena in 26 CVID patients analyzed (8). The present study represents a continuation of these previous studies on the Danish CVID cohort aiming at identifying gene variants with a certain, possible, or potential disease-causing role in CVID patients. Here, we performed whole exome sequencing on a total of 20 CVID patients with autoimmunity, autoinflammation, and/or malignancy and identified both known and novel gene variants with possible/probable disease-causing potential in nine of the patients.

## Methods

### Patient Selection and Inclusion

Patients with a diagnosis of CVID in the Department of Infectious Diseases, Aarhus University Hospital, were included under the following criteria:

A clinical phenotype of sinopulmonary infections and an autoimmune, inflammatory or malignant phenotype. More specifically, patients had decreased IgG and IgM and/or IgA, insufficient vaccine responses to polysaccharide vaccination (when assessed) or reduced isohemagglutinins, and varying degrees of abnormal B-cell phenotype, particularly reduced isotype-switched memory B cells, on flow cytometric analysis. Out of a population of all CVID patients in our clinic—in total 62, we focused on those with a complicated CVID phenotype, that is, those not within the “infectious-only” CVID phenotype with clinical signs and symptoms of autoimmunity, inflammation, granulomatous disease, and/or malignancy. We identified and included a total of 20 patients fulfilling these criteria in the present study.

### Ethics

The study was approved by the Danish Ethics Committee (#1-10-72-354-13). All patients provided informed written consent to participate in the study.

### Whole Exome Sequencing

TruSeq DNA sample preparation was performed according to the manufacturer's recommendations (Illumina), targeting of exomes with EZ Human Medexome Plus Library v3.0 (Roche). Libraries were quantified employing KAPA v.3.13 quantification (Kapabiosystems), and sequencing was performed on NextSeq2, paired end 2X150 bp indexed. Adapters were identified and removed; reads were mapped to hg19 employing BWA mem. PCR and optical duplicates were identified and marked. The alignment file (bam) was re-aligned, employing GATK to refine the alignment. The alignment was recalibrated using GATK. Single nucleotide polymorphisms were called employing HaplotypeCaller from the GATK package. Variant call files (VCF) were uploaded to Ingenuity Variant Analysis and filtered.

### Filtering Strategy/Targeted Gene Screening

Total variants are filtered for confidence (kept variants with call quality at least 20.0 and with allele fraction of at least 25.0) for deleteriousness [kept variants that are frameshift, in-frame indel, or stop codon change, missense, and predicted deleterious by having combined annotation dependent depletion (CADD) score > 15 or within two bases into intron], filtered for presence in a comprehensive list of genes known to be involved in PID or in relevant pathways (list of genes in Supplementary Material 1), and finally excluded variants that are observed with an allele frequency ≥0.01% (heterozygous) or 1% (homozygous variants) of the genomes in the 1,000 genomes project, the NHLBI ESP exomes (European American), the ExAC European Frequency, or the gnomAD European Frequency; unless established Pathogenic common variant (IVA definition). Variants were exported and manually filtered by excluding variants with combined annotation dependent depletion (CADD) score below the mutation significance cutoff (MSC) and manual check of variant in IGV. Furthermore, genome-wide association studies have identified a few SNPs associated with CVID (16–18), and we searched for these in the data. Of the reported variants, only two are present in exons and only one (rs8056264) is sufficiently covered by whole exome sequencing (WES); this variant is not identified, thus the GWAS data cannot be verified in this study.

### Flow Cytometry on B Cells and T Cells

For each analysis, 100 μl heparinized peripheral blood was washed twice in phosphate-buffered saline by centrifugation (5 min at 500 g) before cell labeling with florescent-coupled monoclonal murine anti-human antibodies. Antibodies used for B-cell phenotyping: Anti-CD21[PE-Cy7] (BioLegend/Nordic BioSite, Cat: 354912), -IgD[PE] (Invitrogen/ThermoFisher, Cat: 12-9868-42), -CD19[Super Bright 600] (ThermoFisher, Cat: 63-0198-42), -CD27[VioBright FITC] (Macs Miltenyi Biotec, Cat: 130-113-634), and -CD38[BV421] (BioLegend/Nordic BioSite, Cat: 303526). Antibodies used for T-cell phenotyping: Anti-CD3[APC-Cy7] (BioLegend/Nordic BioSite, Cat: 344818), -CD8a[BV785] (BioLegend/Nordic BioSite, Cat: 301046), -CD27[BV421] (BioLegend/Nordic BioSite, Cat: 302824), -CD4[BV570] (BioLegend/Nordic BioSite, Cat: 300534), -CD197(CCR7)[PE] (BioLegend/Nordic BioSite, Cat: 353204), and -CD45RA[APC] (BioLegend/Nordic BioSite, Cat: 304112). After 30 min incubation, leukocytes were fixed, and red blood cells were lysed by addition of BD FACS Lysing Solution (BD Biosciences). Remaining cells were washed once in PBS, resuspended in PBS, and then analyzed by multicolor flow cytometry (BD FACSCanto, BD Biosciences or NovoCyte 3000, ACEA Biosciences, CA, USA). Matrices for compensation were generated monthly (using BD CompBeads together with the relevant fluorescent-coupled antibodies) or whenever instrument configurations were altered. Data were analyzed using FlowJo software (version 9.7.6, FlowJo LLC, Ashland, OR, USA) or NovoExpress Software (ACEA Biosciences). Lymphocyte phenotyping was based on recommendations (19, 20). Absolute concentrations of blood T cells were determined by flow cytometry for CD3^+^ cells in a lysis-no-wash approach in BD Truecount tubes (FACSCanto) or by direct volumetric flow cytometry (NovoCyte). Concentrations of the additional lymphocyte subsets were calculated from their frequency relative to the CD3^+^ population.

## Results

### Overall Results

Through a targeted whole exome sequencing approach of a subgroup of CVID patients with an autoimmune, inflammatory, or malignant clinical presentation, we sought to identify novel as well as previously described gene variants involved in CVID pathogenesis. Within this cohort of 20 patients, two patients had idiopathic thrombocytopenic purpura (P17 and P18), six had been diagnosed with malignancy, three of which were diffuse large B cell lymphoma (P1, P6, P12), one was splenic marginal zone lymphoma (P17), one was cervical adenocarcinoma (P5), and one was esophagus squamous cell carcinoma (P4) (Table 1). Splenomegaly was described in 13/20 patients (Table 1). All patients received immunoglobulin substitution therapy, except for one (due to anaphylaxis), and 13/20 received additional prophylactic antibiotics, mostly azithromycin (Table S3). Finally, the large majority of patients (with the exception of P15) displayed a markedly reduced fraction of isotype-switched memory B cells by flow cytometry (Table S4).

**Table 1:** Clinical phenotype of patients.

**Patient ID**	**Sex**	**Clinical diagnosis**	**Age at diagnosis**	**Recurrent infectious manifestations**	**Other conditions**	**Hematology**	**Malignancies**
P1	M	CVID	37	Sinuitis	Enteropathy	Trombocytopenia, splenomegaly	Diffuse large B-cell lymphoma
P2	F	CVID	41	Sinuitis	Myelitis (transverse), chronic rhinosinuitis, collagen colitits atopia	Splenomegaly	
P3	M	CVID	35	Pneumonia	Enteropathy, musculoskeletal symptoms	Trombocytopenia, splenomegaly	
P4	M	CVID	50	Pneumonia, warts	Bronchiectasis	Splenomegaly	Esophagus cancer (squamous cell carcinoma)
P5	F	CVID	31	Otitis, sinusitis, pneumonia, fungal infections, warts, abcess	Asthma, bronchiectasis, coeliac disease, lactose intolerance, atopia	Anemia, trombocytopenia, splenomegaly	Cervical cancer (adenocarcinoma)
P6	M	CVID	22	Sinuitis, pneumonia	Bronchiectasis, coeliac disease, lactose intolerance, malabsorption, atopia	Trombocytopenia, splenomegaly	Diffuse large B-cell lymphoma
P7	M	CVID	33	Sinusitis, pneumonia	Elevated liver enzymes, atopia	Trombocytopenia, splenomegaly	
P8	F	TRAPS/gayuhypogammaglobulinaemia, atypical	21	Otitis, sinusitis warts	Malabsorption, lactose inteolerance		
P9	F	CVID	45	Sinusitis, pneumonia	Bronchiectasis, granulomatous disease, atopia	Trombocytopenia, splenomegaly	
P10	F	CVID	23	Pneumonia, stomatitis	Elevated liver enzymes	Anemia, trombocytopenia, splenomegaly (Splenectomized)	
P11	F	CVID	22	Pneumonia	Enteropathy, musculoskeletal symptoms, atopia		
P12	M	CVID	59	Sinusitis, pneumonia, perianal abcess	Enteropathy, gut lymphoma	Splenomegaly	Diffuse large B-cell lymphoma
P13	M	CVID	36	Pneumonia	Enteropathy	Anemia, splenomegaly	
P14	F	CVID	11	Sinusitis, pneumonia	Epilepsia, enteropathy	Trombocytopenia	
P15	F	CVID	27	Sinuitis	Multiple sclerosis, enteropathy (mb. Crohn), autoimmune pancreatitis, atopia		
P16	F	CVID (?)	48	Sinusitis, pneumonia abscesses (mammae and skin)	Asthma, enteropathy, musculoskeletal symptoms, atopia	Lymphocytosis	Cervical dysplasia
P17	F	CVID	58	Sinusitis, pneumonia	Bronchiectasis, neurinoma, enteropathy	Trombocytopenia, splenomegaly	Splenic marginalzone lymphoma
P18	M	CVID	34	Pneumonia, pneumoccocal meningitis		Anemia, trombocytopenia, splenomegaly	
P19	F	CVID	40	Sinusitis		Anemia, trombocytopenia, splenomegaly	
P20	M	HIES (?)	41	Pneumonia	Pulmonal granulomas asthma, atopia		

**Figure 1:**
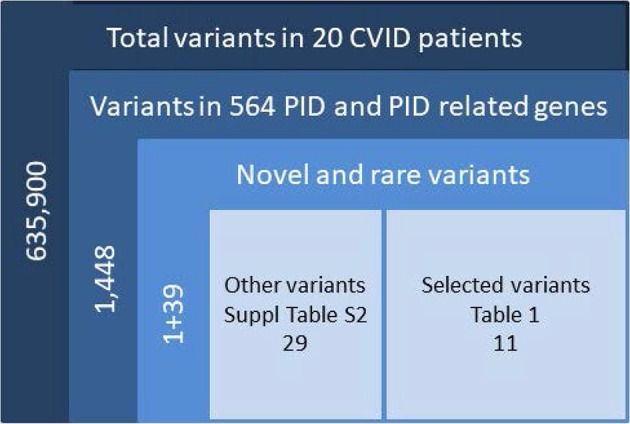
Filtering strategy. Total variants were filtered for confident variants. Included were variants in the exons ±2 bases into introns, either frameshift, in frame indel, stop/start codon change, missense or splice-variants <0.1% for homozygous and <0.01% for heterozygous, further filtered for CADD > MSC and manual check of variants in IGV. CADD, combined annotation-dependent depletion; MSC, mutation significance cutoff; IGV, Ingenuity Variant analysis.

We sequenced 20 adult patients, and the average age of the cohort was 46 years (range: 25–64 years), and the distribution of females:males was 11:9. A total of 635,900 variants were identified in patient exomes. Variants were filtered based on call quality of 20.0, allele frequency above 25%, and deleteriousness to identify significant variants within a comprehensive list of 564 immune deficiency and immune pathway-related genes (Table S1), resulting in 1,448 variants (Figure 1). Of these 40 variants in 17 patients were novel or rare with a combined annotation depletion-dependent (CADD) score above the MSC, 11 variants in nine patients (45%) were considered possibly disease-causing (Table 2) while the remaining 29 variants in 15 patients were considered unlikely disease-causing (Table S2). Of the 11 described variants, four were classified as likely pathogenic according to ACMG guidelines, whereas the remaining were classified as variants of unknown significance (Table 2). One of the variants identified was segregating in a family, whereas none of the remaining variants were known familial (Table 3). Novel variants in *NFKB1, STAT3, TNFAIP3*, and *TTC37* and rare variants in *IL-17F, IRAK4, DDX41, NLRC3, TNFRSF1A*, and *PLCG2* were identified (Table 2). The remaining variants are listed in Table S2. Additional information is available in the following tables: Clinical phenotype of patients (Table 1), Results of clinical immunological evaluation (Table 4), Additional clinical and paraclinical results and treatment (Table S3), Immunophenotyping of B-cell subsets (Table S4), and T-cell subsets (Table S5).

**Table 2:** Monoallelic variants identified.

**Patient ID**	**Gene symbol**	**Transcript ID**	**Transcript variant**	**Protein variant**	**gnomAD freq**.	**SIFT**	**PolyPhen-2**	**CADD score**	**MSC**	**ACMG classification**
P2	*NFKB1*	NM_003998.3	c.950_964del CAAAGTATAAAGATA	p.P317_I322delinsL					3.313	4
P2	*NFKB1*	NM_003998.3	c.967A>T	p.N323Y	-	D	B	24.5	3.313	4
P3	*STAT3*	NM_139276.2	c.319C>T	p.R107W	-	T	PrD	25.2	15.290	3
P10	*TNFAIP3*	NM_006290.3	c.997dupG	p.A333fs^*^2	-				3.313	4
P11	*IL17F*	NM_052872.3	c.254C>T	p.T85I	0.002	T	B	22.5	3.313	3
P12	*IRAK4*	NM_016123.3	c.1120G>A	p.G374S	0.001	D	PrD	32	0.001	3
P15	*TTC37*	NM_014639.3	c.3988_3989delTC	p.S1330fs^*^14	-				4.836	4
P16	*DDX41*	NM_016222.3	c.1435C>T	p.R353W	0.002	D	PrD	29.8	3.313	3
P17	*NLRC3*	NM_178844.3	c.1189G>A	p.V397M	0.001	T	B	15.9	3.313	3
P18	*TNFRSF1A*	NM_001065.3	c.1343C>T	p.P448L	0.002	T	B	15	0.015	3
P18	*PLCG2*	NM_002661.4	c.2866C>T	p.R956C	0.002	T	PrD	33	3.313	3

*D, damaging; T, Tolerated; B, Benign; PoD, Possibly Damaging; PrD, Probably Damaging*.

*Referencer: CADD, combined annotation dependent depletion, PMID 24487276; PolyPhen-2: PMID 20354512; SIFT: PMID 19561590; MSC, mutation significance cutoff, https://doi.org/10.1038/nmeth.3739; ACMG, American College of Medical Genetics and Genomics, https://doi.org/10.1038/gim.2015.30*.

**Table 3:** Gene function and associated clinical phenotype of identified gene variants.

**Patient ID**	**Gene symbol**	**Gene function**	**Associated clinical phenotype**	**Inheritance**	**References**
P2	*NFKB1*	Transcription factor	AD Common variable immunodeficiency 12	*De novo*	(21)
P2	*NFKB1*				
P3	*STAT3*	Signal transducer and transcription activator	AD Hyper-IgE recurrent infection syndromegayu AD Autoimmune disease, multisystem, infantile-onset, 1	From healthy father, seen in diseased daugther	(22)
P10	*TNFAIP3*	Zinc finger protein and ubiqitin-editing enzyme	AD Autoinflammatory syndrome, Behcet-like	Unknown	(23, 24)
P11	*IL17F*	Cytokine	AD candidiasis	Unknown	(25)
P12	*IRAK4*	Serine/threonine-protein kinase	AR IRAK4 deficiency	Unknown	(26)
P15	*TTC37*	Involved in exosome-mediated RNA decay^*^	AR Trichohepatoenteric syndrome 1	Segregates with disease	(27)
P16	*DDX41*	ATP-dependent RNA helicase^*^	AD Myeloproliferative/lymphoproliferative neoplasms	Unknown	(28)
P17	*NLRC3*	Negative regulator of the innate immune response	None reported yet	Unknown	
P18	*TNFRSF1A*	Receptor for TNFSF2/TNF-alpha	AD Periodic fever	Unknown	(29)
P18	*PLCG2*	Phospholipase	AD Autoinflammation, antibody deficiency, and immune dysregulation syndromegayu Familial cold autoinflammatory syndrome 3		(30, 31)

AD, autosomal dominant; AR, autosomal recessive; XLD, X-linked;

* *Probable function*.

**Table 4:** Clinical immunology results.

**Patient ID**	**IgG^#^ (g/L)**	**IgM (g/L)**	**IgA (g/L)**	**Lymfocyte count (cells/μL WB)**	**B cells (CD19+; cells/μL)**	**NK cells (CD56+; cells/μL)**	**T cells (CD3+; cells/μL)**	**CD4 (cells/μL)**	**CD8 (cells/μL)**	**CD4/CD8-ratio**	**Isohaemagglutinin IgM/IgG**
P1	2.9	<0.2	BD	881	63	29	774	290	435	0.67	NP
P2	2.9	0.32	BD	5,800	160	40	1,200	800	350	2.4	NP
P3	1.1	<0.2	BD	908	68	109	717	430	230	1.88	NP
P4	0.3	<0.2	BD	2,140	738	257	1,119	693	393	1.76	NP
P5	0.9	<0.2	BD	400	2	46	347	155	153	1.01	After IVIG/after IVIG
P6	^*^	<0.2	BD	493	59	79	281	143	109	1.32	After IVIG/after IVIG
P7	^*^	<0.2	BD	632	15	58	504	215	274	0.79	NP
P8	4.4	0.59	0.7	1,072	77	93	889	530	264	2.01	Normal
P9	^*^	<0.2	BD	324	22	25	270	186	74	2.51	NP
P10	4.2	^*^	BD	266	68	34	156	102	48	2.13	NP (only after IVIG)
P11	^*^	<0.2	BD	1,147	92	203	848	240	522	0.46	NP
P12	3.3	0.3	BD	516	118	94	285	168	100	1.67	NP
P13	0.47	<0.2	BD	1,323	106	118	1,050	431	562	0.77	Absent A; decreased (B) (both IgM+IgG)
P14	^*^	<0.2	BD	1,600	224	51	1,312	853	354	2.41	NP
P15	5.9	0.82	0.54	2,917	268	76	2,482	1,633	730	2.24	Decreased/decreased
P16	4.8	0.42	1.58	3,213	321	112	2,757	1,566	1,023	1.53	Decreased/decreased
P17	0.7	0.15	BD	1,877	135	139	1,541	925	564	1.64	Decreased/decreased
P18	2.2	1.12	0.13	5,113	1,089	547	3,288	1,772	1,065	1.66	Decreased/normal
P19	4.3	0.49	0.24	1,600	149	210	1,182	876	234	3.74	Decreased/decreased
P20	7.5	0.23	3.45	249	18	106	123	97	20	4.81	NP

* *No measurement available*;

#, *IgG before initiation of immunoglobulin substitution therapy; BD, below detection limit. Reference values: IgG_tot_ (6.9–15.7 g/L); IgM (g/L) (0.55–3.9); IgA (g/L) (BD; below detection limit) (0.8–3.9); Lymfocyte count (cell/μL WB) (1,000–2,800); B cells (CD19+; cells/μL) (70–272); NK cells (CD56+; cells/μL) (52–639); T cells (CD3+; cells/μL) (581–1,895); CD4 (cells/μL) (309–1,140); CD8 (cells/μL) (137–823); CD4/CD8-ratio (PMID: 14962239); NP, not performed; BD; below detection limit*.

### Identified Variants

P2, was a 50-year-old woman with transverse myelitis, chronic rhinosinusitis, collagen colitis, musculoskeletal symptoms, splenomegaly, atopia, and infections (Table 1). A deleterious heterozygous 15 nucleotide (nt) deletion (p.P317_I322delinsL) and missense (p.N323Y) mutation in the nuclear factor-κB (*NFKB)1* gene were identified (Tables 2, 3). Both *NFKB1* variants are positioned on the same allele in the Ig-like, plexins, transcription factors (IPT) domain, and are predicted to have an impact on the function of the protein. The mutation was verified by Sanger sequencing, and it was *de novo*—the healthy parents did not carry the variant neither did a healthy daughter. Other variants in *NFKB1* in CVID have been described as the most common cause of CVID with non-infectious complications, including lymphadenopathy, splenomegaly, and autoimmune disease, and all reported patients exhibit deficient B-lymphocyte differentiation with increased CD21low B-cell numbers (21, 32). Finally, the observed/expected (o/e) ration of 0.04 indicates strong selection against loss-of-function (LoF) variants in *NFKB1* (33).

P3, a 41-year-old male, presented with thrombocytopenia, splenomegaly, lymphadenopathy, enteropathy, and a single pneumonia (Table 1). The variant is inherited from the healthy father and also carried by the sister, who suffered from Crohn's disease (Table S3). He carried a signal transducer and activator of transcription (*STAT)3* variant (p.R107W) (Tables 2, 3) predicted to be deleterious, and there is some selection against missense variants in STAT3 (0/e = 0.62). The variant has not previously been reported. It is localized in the N-terminal domain, in which gain-of-function (GOF) *STAT3* variants have been described (22). A recent overview of patients with STAT3 GOF describes the general phenotype of the patients, including autoimmune cytopenias, lymphadenopathy, enteropathy, and interstitial lung disease, while immunodeficiency is not predominant (34).

Patient 10 was diagnosed with CVID 10 years ago and experienced severe disease with significant hepatosplenomegaly, pancytopenia with persistent CD4 lymphopenia, and pneumonias. Liver biopsy showed nodular regenerative hyperplasia and esophagoscopy revealed portal hypertension and esophagus varices documented by esophagoscopy. Splenectomy relieved some of the hypersplenism and improved thrombocytopenia, and the patient moreover received granulocyte colony-stimulatory factor (G-CSF) to avoid neutropenia. A variant in the tumor necrosis factor, alpha-induced protein (*TNFAIP)3* gene (c.997dupG) resulting in a frameshift (p.A333fs^*^2) was found in P10 (Tables 2, 3). The *TNFAIP3* gene encodes a zinc finger protein and ubiquitin-editing enzyme, and variants in *TNFAIP3* have been associated with autoinflammatory syndrome (OMIM #616744) characterized by hemolytic anemia, thrombocytopenia, autoinflammation, and autoimmune lymphoproliferative syndrome, the latter presenting with lymphadenopathy and hepatosplenomegaly (23). The variant harbored by P10 has not been described previously, and we have not been able to verify whether the variant is *de novo* or inherited; however a p.A332X mutation has been reported in autosomal dominant familial Behçet disease and haploinsufficiency of A20 (35). The variant in P10 is located in the ovarian tumor (OUT) domain as described for p.A332X, resulting in a severely truncated and rapidly degraded protein (35). The clinical presentation of the patient shares many features with A20 haploinsufficiency.

In P11, a 32-year-old female patient diagnosed with CVID at the age of 22 and with pneumonias and gastrointestinal symptoms, a novel variant in the interleukin (*IL)17F* gene, was identified (Tables 2, 3). The patient is heterozygous for this rare missense variant, p.T85I, not previously reported in relation to any disease and not present in 60,000 control samples [gnomAD v2.1.1(controls)]. The o/e ratio of missense variants in the *IL17F* gene (1.05) however indicates that selection against missense mutations is not strong. Nevertheless, variants in the *IL17F* cytokine gene have been reported in autosomal dominant candidiasis with a hypomorphic S65L allele (25). However, the patient reported here was described with bacterial infections and without any story of recurrent candida infections or staphylococcal abscesses.

P12 was a man with marked complications, including pneumonias, sinusitis, enteropathy, and more recently a diffuse large B cell lymphoma in the gut. A rare potentially damaging missense variant was identified in the interleukin-1 receptor-associated kinase *(IRAK)4* gene encoding Interleukin-1 receptor-associated kinase 4 (Tables 2, 3). The missense variant p.G374S, which is not present in 60,000 control samples [gnomAD v2.1.1(controls)], is located in the kinase domain. IRAK4 is a serine/threonine-protein kinase activator of NF-κB. Generally, IRAK4 deficiency is caused by homozygous or compound heterozygous *IRAK4* deficiency, resulting in predisposition to recurrent invasive pyogenic bacterial infection in children (26). The o/e ratio of the missense variants in the *IRAK4* gene (0.96) however indicates that selection against missense mutations is not strong. However, a potential role of a heterozygous variant working by haploinsufficiency or dominant negative effect in this patient cannot be fully excluded.

P15 was a 34-year-old patient diagnosed with “atypical CVID” at the age of 26 and with a history of hypogammaglobulinemia, multiple sclerosis, recurrent miscarriages, and inflammatory bowel disease (Mb. Crohn) with an episode of autoimmune pancreatitis. A novel likely deleterious frameshift S1330fs^*^14 variant in tetratricopeptide repeat domain (*TTC)37* was found in P15 (Tables 2, 3). Her symptoms were only slightly overlapping with autosomal recessive Tricho-Hepato-Enteric Syndrome (OMIM #222470), which is characterized by intrauterine growth retardation, wooly hair, facial dysmorphism, intractable diarrhea in infancy, and immunodepression, previously associated with mutations in this gene (27). However, some patients have been described with only one mutation, and therefore a weak phenotype might be caused by a heterozygous variant. The o/e ratio of LoF variants in the *TTC37* gene (0.63) does indicate some selection against LoF. The variant was also detected in the mother, a brother and a sister, all with similar symptoms as P15, whereas two healthy brothers and the healthy father did not carry the variant.

P16, a 48-year-old woman, suffered from cervical dysplasia and lymphocytosis. In P16, a possibly deleterious variant in DEAD-Box Helicase (*DDX)41* (p.R353W), not present in 60,000 control samples [gnomAD v2.1.1(controls)] was present (Tables 2, 3). *DDX41* encodes an ATP-dependent RNA helicase and variants predispose to myeloproliferative/lymphoproliferative neoplasms (OMIM #616871) (28) and thus may be related to cervical dysplasia in the patient. Some selection against missense variants is indicated by the o/e ratio of missense variants in the *DDX41* gene (0.68).

P17, a 58-year-old woman, had a clinical history including bronchiectasis, recurrent pneumonias, neurinoma, enteropathy, elevated liver enzymes, atopia, idiopathic thrombocytopenia, and splenomegaly (Table 1). A potentially deleterious variant in NOD-like receptor family CARD domain containing (*NLRC)3* c.1189G > A; p.V397M was identified (Tables 2, 3). Variants in *NLRC3* have not previously been reported in association with disease, although lack of NLRC3 has been shown to result in increased activation of NF-κB and elevated production of pro-inflammatory cytokines following lipopolysaccharide (LPS) stimulation in mice (36). The variant identified in P17 is located in the NACHT domain of NLRC3 and predicted to be deleterious, whereas selection against missense variants is in general not observed (o/e ratio = 0.99).

Finally, in P18, a 40-year-old male with idiopathic thrombocytopenia in childhood and severe pneumococcal meningitis at the age of 34 years, two variants in *PLCG2* and *TNFRSF1A* were identified (Tables 2, 3), The specific variant, *PLCG2* p.R956C, is rare and potentially harmful with a high CADD score of 35 but not previously described in association with disease (Tables 2, 3), and selection against missense variants seems to be low (o/e = 0.92). Variants in the *PLCG2* gene have been associated with autosomal dominant autoinflammation, antibody deficiency, and immunodysregulation syndrome (OMIM #614878) (30, 31). The variant in *TNFRSF1A* is rare, potentially damaging and variants in this gene have been reported to cause autosomal dominant periodic fever syndromes; there is some selection against missense variants in *TNFRSF1A* (o/e = 0.64) (24, 29). Altogether, the patient history is more immediately related to a potential PLCG2 defect than to autoinflammatory periodic fever associated to abnormal function of TNFRSF1A.

## Discussion

In this study, we performed WES on a selected subgroup of CVID patients with a classical infectious phenotype combined with autoimmunity, inflammation, and/or malignancy and identified probable and possible disease-causing candidate gene variants through a targeted bioinformatics analysis, focusing on 564 primary immunodeficiency-related genes. This approach resulted in the identification of two previously described gene variants in *NFKB1* (21, 32), nine novel variants, including variants in *STAT3, TNFAIP3, IL-17F, IRAK4, TTC37, DDX41, NLRC3, TNFRSF1A*, and *PLCG2* (22–31, 34–36) associated with different states of immune dysregulation but not previously associated with CVID, and with possible probable disease-causing impact according to bioinformatics. Altogether, we identified probable/possible disease-causing variants in nine of 20 patients in this CVID subpopulation. While this is a high proportion compared to previous studies on genetic etiologies of CVID, this number may not be surprising, given that this was a selected population of “complicated CVID patients,” representing roughly one third of the total number of CVID patients in our clinic in a university hospital.

Over the past few years, with the advent and introduction of next-generation sequencing, not only for research purposes but into clinical practice, data on genetic variants and their disease-causing potential in CVID are accumulating. However, the genetic basis of CVID appears to be highly complex, and many aspects of disease pathogenesis need to be further explored (37). The aim of performing genetic analysis in CVID patients is to gain a better fundamental understanding of disease pathogenesis and also to help stratifying patients into disease entities to predict disease complications and prognosis and to possibly individualize treatment. Genetic analysis of CVID patients has established the involvement of bi-allelic mutations in *ICOS, CD19, CD20, CD21, CD80, PKCD*, and *LRBA*. Furthermore, mono-allelic dominant mutations have been identified in *PIK3CD, NFKB2, PIK3R1 NFKB1*, and *IKZF1* (38). Most genes identified in CVID patients so far are thus encoding molecules with cellular functions as receptors or signaling components in B-cell development, differentiation, activation, and homeostasis (1, 38). More recent data have revealed a major role for disturbances in the NF-κB pathway in some CVID patients (39). Importantly, this more detailed molecular and immunological analysis has also revealed that some of the fractions within the population of “CVID patients” should be classified separately and be regarded rather as combined immunodeficiencies due to defects in interactions between T cells and B cells, for instance in the case of cytotoxic T lymphocyte-associated antigen (CTLA)4 deficiency and LPS-responsive and beige-like anchor protein (LRBA) deficiency (40, 41).

Recently a study very similar to the present one was published, in which a cohort of 50 non-selected CVID patients were exome sequenced and variants were selected under very similar conditions as we used in our study (38). The publication by Maffucci et al. reported the identification of 17 probable disease-causing mutations in 15 patients, altogether representing variants in about 30% of patients. The identified variants included mono-allelic mutations in *NFKB1, STAT3, CTLA4, PIK3CD*, and *IKZF1*, and bi-allelic mutations in *LRBA* and *STXBP*2 (38), and the authors concluded that WES combined with analysis of PID-associated genes is a cost-effective way to identify variants with potential benefits for risk stratification and targeted treatment for certain subgroups of CVID patients.

A major conclusion from the present study is that surprisingly few of the previously identified genetic variants in populations of CVID patients were found in our analysis, since indeed most variants identified and reported here were novel. In some cases, other variants in the same molecule have even been associated with other PIDs, such as chronic mucocutaneous candidiasis for *IL-17F*, recurrent pyogenic infections for *IRAK4*, and autoinflammatory fever syndromes for *TNFAIP3, TNFRSF1A*, and *PLCG2*, although mostly in patients homozygous for the given variant. These data indicate that CVID and possibly other PIDs may be caused by mono-allelic variants in genes otherwise known to cause a distinct clinical phenotype when patients are homozygous or compound heterozygous for these variants. Moreover, quite diverse clinical pictures may arise, depending on whether a disease-causing variant is LOF to cause immunodeficiency, or alternatively gain-of-function to cause autoinflammation. Paradoxically, some of the gene variants in CVID appear to cause immunodeficiency together with autoinflammation in certain cases. This underscores the complexity of studying the genetics of CVID and suggest that genetic variants in a relatively large number of genes may play a role in CVID pathogenesis (37). However, these findings are in line with accumulating data and experience from other PIDs, where a common theme that emerges is that different variants in a given molecule may give rise to significantly different disease presentations and major differences in infection susceptibility. The limited number of patients included in the present study hampers the possibility to draw any firm conclusions on specific genetic variants associated with CVID with autoinflammation vs. CVID associated with malignancy. Future larger studies should build on these data and the literature available to pursue these questions.

One possible weakness of our study is that the selection of a particularly complex disease phenotype will overestimate the true fraction of disease-causing gene variants in CVID patients. However, here we wished to focus on genes associated with the more autoimmune-inflammatory-malignant phenotype and thereby potentially increase knowledge on the disease pathogenesis in this patient group, as well as increasing the likelihood to identify disease-causing mutations and thereby improve the quality of the WES bioinformatics analysis and the conclusions drawn from it. Therefore, this study should be interpreted with these cautions and clearly reflects the situation in this one third of CVID patients with a complex CVID phenotype.

Moreover, performing WES to identify disease-causing variants, it is always the gold standard and optimal situation to functionally validate identified variants, particular those involving new gene variants as well as those localized in new positions of genes previously associated to disease (42, 43). Indeed a number of criteria were suggested as an optimal gold standard when claiming a disease-causing potential of a gene. These criteria include (1) bioinformatics prediction of deleteriousness of the variant, (2) evolutionary conservation/positive selection, (3) similar rarity/frequency of the variant in the general population as the disease under study, (4) reconstitution of the wild type (WT) by reintroduction of the WT gene in patient cells, (5) a known role of the gene in immunological/cell biological processes relevant for disease pathogenesis, and (6) potentially mimicking of the patient disease phenotype in a relevant cell system or animal model (42). Although none of the criteria is absolutely obligate, they each contribute to the likelihood of identifying relevant disease-causing variants. Likewise, none of the criteria can exclusively establish this relationship. In the present study, we were not able to functionally validate identified variants *in vitro* or *in vivo*, but we did select variants based on the criteria related to bioinformatics prediction of deleteriousness, rarity, and relevance in disease pathogenesis, in this case CVID with autoimmunity/autoinflammation. We therefore strongly suggest that the variants identified in the present work should lay the ground for future studies to functionally validate their disease-causing potential and to investigate at the mechanistic and molecular level their precise role in CVID pathogenesis.

Understanding an increasing number of genetic mechanisms associated with the complex CVID phenotype has also pointed to the need for a new view on this disease and has suggested that it may be appropriate to consider new classification schemes, for instance dissecting combined immunodeficiencies from “true mainly B cell-based CVID.” However, only for 25–30% of CVID patients, a certain/probable genetic etiology has been established. This number is, however, likely to increase over the coming years as a growing number of patients are undergoing sequencing. Finally, CVID may also have a digenic or polygenic basis, which is more complicated to establish with current sequencing tools and bioinformatics. Future studies are needed to more conclusively establish how genetic analysis of CVID patients should be integrated into clinical practice; which specific genetic variants increase the risk of autoimmunity, autoinflammation, and malignancy; and how this knowledge should be translated into improved antibiotic prophylaxis, immunomodulatory treatment of autoimmune/autoinflammatory complications, and possibly intensified screening programs for malignancy. We believe that the present work contributes with important new insights into the genetic basis of CVID and particular in the subset of CVID patients with a complex phenotype involving not only infection, but also autoimmunity, autoinflammation, and malignancy. This should help increase insight into the pathogenesis of this intriguing disease and at the same time improve clinical management of patients with CVID.

## Data Availability Statement

All datasets generated for this study are included in the article/Supplementary Material.

## Ethics Statement

The studies involving human participants were reviewed and approved by the Danish National Committee on Health Research Ethics. The patients/participants provided their written informed consent to participate in this study.

## Author Contributions

TM, CL, and MC conceived the idea and planned the study. TM and CL identified and cared for patients. MC performed analysis of WES data. RO collected clinical and immunological data on patients (disease presentation, blood analyses, imaging, and clinical immunological evaluation). MP and JJ performed flow cytometry to determine immune cell phenotyping. MC and TM drafted the first version of the manuscript. All authors read, commented, and approved the final version of the manuscript.

### Conflict of Interest

The authors declare that the research was conducted in the absence of any commercial or financial relationships that could be construed as a potential conflict of interest.

## References

[1] Yong PF Thaventhiran JE Grimbacher B A rose is a rose is a rose, but CVID is Not CVID common variable immune deficiency (CVID), what do we know in 2011? Adv Immunol. (2011) 111:47–107. 10.1016/B978-0-12-385991-4.00002-7 21970952

[2] Westh L Mogensen TH Dalgaard LS Bernth Jensen JM Katzenstein T Hansen AE. Identification and characterization of a nationwide danish adult common variable immunodeficiency cohort. Scand J Immunol. (2017) 85:450–61. 10.1111/sji.12551 28370285

[3] Wehr C Kivioja T Schmitt C Ferry B Witte T Eren E. The EUROclass trial: defining subgroups in common variable immunodeficiency. Blood. (2008) 111:77–85. 10.1182/blood-2007-06-091744 17898316

[4] Resnick ES Cunningham-Rundles C. The many faces of the clinical picture of common variable immune deficiency. Curr Opin Allergy Clin Immunol. (2012) 12:595–601. 10.1097/ACI.0b013e32835914b9 23026770

[5] Resnick ES Moshier EL Godbold JH Cunningham-Rundles C. Morbidity and mortality in common variable immune deficiency over 4 decades. Blood. (2012) 119:1650–7. 10.1182/blood-2011-09-377945 22180439 PMC3286343

[6] Carter CR Aravind G Smalle NL Cole JY Savic S Wood PM. CVID patients with autoimmunity have elevated T cell expression of granzyme B and HLA-DR and reduced levels of Treg cells. J Clin Pathol. (2013) 66:146–50. 10.1136/jclinpath-2012-201046 23172556

[7] Sweinberg SK Wodell RA Grodofsky MP Greene JM Conley ME. Retrospective analysis of the incidence of pulmonary disease in hypogammaglobulinemia. J Allergy Clin Immunol. (1991) 88:96–104. 10.1016/0091-6749(91)90306-9 2071789

[8] Kofod-Olsen E Jorgensen SE Nissen SK Westh L Moller BK Ostergaard L. Altered fraction of regulatory B and T cells is correlated with autoimmune phenomena and splenomegaly in patients with CVID. Clin Immunol. (2016) 162:49–57. 10.1016/j.clim.2015.11.003 26586095

[9] Maglione PJ Cols M Cunningham-Rundles C. Dysregulation of innate lymphoid cells in common variable immunodeficiency. Curr Allergy Asthma Rep. (2017) 17:77. 10.1007/s11882-017-0746-6 28983810 PMC5897894

[10] Arala-Chaves MP Korn JH Galbraith GM Porto MT Smith CL Fudenberg HH. Effects of thymosin and evidence of monocyte suppression of both T- and B-cell functions in two cases of 'common variable immunodeficiency'. Scand J Immunol. (1982) 15:97–104. 10.1111/j.1365-3083.1982.tb00626.x 6978521

[11] Conley ME Notarangelo LD Etzioni A. Diagnostic criteria for primary immunodeficiencies. Representing PAGID (Pan-American Group for Immunodeficiency) and ESID (European Society for Immunodeficiencies). Clin Immunol. (1999) 93:190–7. 10.1006/clim.1999.4799 10600329

[12] Warnatz K Denz A Drager R Braun M Groth C Wolff-Vorbeck G. Severe deficiency of switched memory B cells (CD27(+)IgM(-)IgD(-)) in subgroups of patients with common variable immunodeficiency: a new approach to classify a heterogeneous disease. Blood. (2002) 99:1544–51. 10.1182/blood.V99.5.1544 11861266

[13] Ameratunga R Woon ST Gillis D Koopmans W Steele R. New diagnostic criteria for CVID. Expert Rev Clin Immunol. (2014) 10:183–6. 10.1586/1744666X.2014.875274 24410535

[14] Bonilla FA Barlan I Chapel H Costa-Carvalho BT Cunningham-Rundles C de la Morena MT. International Consensus Document (ICON): common variable immunodeficiency disorders. J Allergy Clin Immunol Pract. (2016) 4:38–59. 10.1016/j.jaip.2015.07.025 26563668 PMC4869529

[15] Seidel MG Kindle G Gathmann B Quinti I Buckland M van Montfrans J. The European Society for Immunodeficiencies (ESID) registry working definitions for the clinical diagnosis of inborn errors of immunity. J Allergy Clin Immunol Pract. (2019) 7:1763–70. 10.1016/j.jaip.2019.02.004 30776527

[16] Perovic D Perovic V Pravica V Bonaci-Nikolic B Mijanovic R Bunjevacki V. Evaluation of cytokine genetic polymorphisms in adult patients with common variable immunodeficiency: a single-center study. Immunol Lett. (2016) 176:97–104. 10.1016/j.imlet.2016.05.005 27288995

[17] Maggadottir SM Li J Glessner JT Li YR Wei Z Chang X. Rare variants at 16p11.2 are associated with common variable immunodeficiency. J Allergy Clin Immunol. (2015) 135:1569–77. 10.1016/j.jaci.2014.12.1939 25678086 PMC4461447

[18] Orange JS Glessner JT Resnick E Sullivan KE Lucas M Ferry B. Genome-wide association identifies diverse causes of common variable immunodeficiency. J Allergy Clin Immunol. (2011) 127:1360–7.e6. 10.1016/j.jaci.2011.02.039 21497890 PMC3646656

[19] Warnatz K Schlesier M. Flowcytometric phenotyping of common variable immunodeficiency. Cytometry B Clin Cytom. (2008) 74:261–71. 10.1002/cyto.b.20432 18561200

[20] Appay V van Lier RA Sallusto F Roederer M. Phenotype and function of human T lymphocyte subsets: consensus and issues. Cytometry A. (2008) 73:975–83. 10.1002/cyto.a.20643 18785267

[21] Fliegauf M Bryant VL Frede N Slade C Woon ST Lehnert K. Haploinsufficiency of the NF- κB1 Subunit p50 in Common Variable Immunodeficiency. Am J Hum Genet. (2015) 97:389–403. 10.1016/j.ajhg.2015.07.008 26279205 PMC4564940

[22] Flanagan SE Haapaniemi E Russell MA Caswell R Allen HL De Franco E. Activating germline mutations in STAT3 cause early-onset multi-organ autoimmune disease. Nat Genet. (2014) 46:812–4. 10.1038/ng.3040 25038750 PMC4129488

[23] Takagi M Ogata S Ueno H Yoshida K Yeh T Hoshino A. Haploinsufficiency of TNFAIP3 (A20) by germline mutation is involved in autoimmune lymphoproliferative syndrome. J Allergy Clin Immunol. (2017) 139:1914–22. 10.1016/j.jaci.2016.09.038 27845235

[24] Zhou Q Wang H Schwartz DM Stoffels M Park YH Zhang Y. Loss-of-function mutations in TNFAIP3 leading to A20 haploinsufficiency cause an early-onset autoinflammatory disease. Nat Genet. (2016) 48:67–73. 10.1038/ng.3459 26642243 PMC4777523

[25] Puel A Cypowyj S Bustamante J Wright JF Liu L Lim HK. Chronic mucocutaneous candidiasis in humans with inborn errors of interleukin-17 immunity. Science. (2011) 332:65–8. 10.1126/science.1200439 21350122 PMC3070042

[26] Picard C Puel A Bonnet M Ku CL Bustamante J Yang K. Pyogenic bacterial infections in humans with IRAK-4 deficiency. Science. (2003) 299:2076–9. 10.1126/science.1081902 12637671

[27] Bourgeois P Esteve C Chaix C Beroud C Levy N consortium Tc. Tricho-Hepato-enteric syndrome mutation update: mutations spectrum of TTC37 and SKIV2L, clinical analysis and future prospects. Hum Mutat. (2018) 39:774–89. 10.1002/humu.23418 29527791

[28] Polprasert C Schulze I Sekeres MA Makishima H Przychodzen B Hosono N. Inherited and somatic defects in DDX41 in myeloid neoplasms. Cancer Cell. (2015) 27:658–70. 10.1016/j.ccell.2015.03.017 25920683 PMC8713504

[29] McDermott MF Aksentijevich I Galon J McDermott EM Ogunkolade BW Centola M. Germline mutations in the extracellular domains of the 55 kDa TNF receptor, TNFR1, define a family of dominantly inherited autoinflammatory syndromes. Cell. (1999) 97:133–44. 10.1016/S0092-8674(00)80721-7 10199409

[30] Zhou Q Lee GS Brady J Datta S Katan M Sheikh A. A hypermorphic missense mutation in PLCG2, encoding phospholipase Cgamma2, causes a dominantly inherited autoinflammatory disease with immunodeficiency. Am J Hum Genet. (2012) 91:713–20. 10.1016/j.ajhg.2012.08.006 23000145 PMC3484656

[31] Ombrello MJ Remmers EF Sun G Freeman AF Datta S Torabi-Parizi P. Cold urticaria, immunodeficiency, and autoimmunity related to PLCG2 deletions. N Engl J Med. (2012) 366:330–8. 10.1056/NEJMoa1102140 22236196 PMC3298368

[32] Tuijnenburg P Lango Allen H Burns SO Greene D Jansen MH Staples E. Loss-of-function nuclear factor κB subunit 1 (NFKB1) variants are the most common monogenic cause of common variable immunodeficiency in Europeans. J Allergy Clin Immunol. (2018) 142:1285–96. 10.1016/j.jaci.2018.01.039 29477724 PMC6148345

[33] Karczewski KJ Francioli LC Tiao G Cummings BB Alföldi J Wang Q Variation across 141,456 human exomes and genomes reveals the spectrum of loss-of-function intolerance across human protein-coding genes. bioRxiv [Preprint]. (2019). 10.1101/531210

[34] Fabre A Marchal S Barlogis V Mari B Barbry P Rohrlich PS. Clinical aspects of STAT3 gain-of-function germline mutations: a systematic review. J Allergy Clin Immunol Pract. (2019) 7:1958–69.e9. 10.1016/j.jaip.2019.02.018 30825606

[35] Berteau F Rouviere B Delluc A Nau A Le Berre R Sarrabay G. Autosomic dominant familial Behcet disease and haploinsufficiency A20: a review of the literature. Autoimmun Rev. (2018) 17:809–15. 10.1016/j.autrev.2018.02.012 29890348

[36] Schneider M Zimmermann AG Roberts RA Zhang L Swanson KV Wen H. The innate immune sensor NLRC3 attenuates Toll-like receptor signaling via modification of the signaling adaptor TRAF6 and transcription factor NF-κB. Nat Immunol. (2012) 13:823–31. 10.1038/ni.2378 22863753 PMC3721195

[37] Ameratunga R Lehnert K Woon ST Gillis D Bryant VL Slade CA. Review: diagnosing common variable immunodeficiency disorder in the era of genome sequencing. Clin Rev Allergy Immunol. (2018) 54:261–8. 10.1007/s12016-017-8645-0 29030829

[38] Maffucci P Filion CA Boisson B Itan Y Shang L Casanova JL. Genetic diagnosis using whole exome sequencing in common variable immunodeficiency. Front Immunol. (2016) 7:220. 10.3389/fimmu.2016.00220 27379089 PMC4903998

[39] Keller B Cseresnyes Z Stumpf I Wehr C Fliegauf M Bulashevska A. Disturbed canonical nuclear factor of κ light chain signaling in B cells of patients with common variable immunodeficiency. J Allergy Clin Immunol. (2017) 139:220–31.e8. 10.1016/j.jaci.2016.04.043 27461466

[40] Schubert D Bode C Kenefeck R Hou TZ Wing JB Kennedy A. Autosomal dominant immune dysregulation syndrome in humans with CTLA4 mutations. Nat Med. (2014) 20:1410–6. 10.1038/nm.3746 25329329 PMC4668597

[41] Lopez-Herrera G Tampella G Pan-Hammarstrom Q Herholz P Trujillo-Vargas CM Phadwal K. Deleterious mutations in LRBA are associated with a syndrome of immune deficiency and autoimmunity. Am J Hum Genet. (2012) 90:986–1001. 10.1016/j.ajhg.2012.04.015 22608502 PMC3370280

[42] Casanova JL Conley ME Seligman SJ Abel L Notarangelo LD. Guidelines for genetic studies in single patients: lessons from primary immunodeficiencies. J Exp Med. (2014) 211:2137–49. 10.1084/jem.20140520 25311508 PMC4203950

[43] Meyts I Bosch B Bolze A Boisson B Itan Y Belkadi A. Exome and genome sequencing for inborn errors of immunity. J Allergy Clin Immunol. (2016) 138:957–69. 10.1016/j.jaci.2016.08.003 27720020 PMC5074686

