# Supplementary material for: Identification of Novel Genetic Variants in CVID Patients With Autoimmunity, Autoinflammation, or Malignancy
Source: Front Immunol. 2020 Jan 27;10:3022. doi: 10.3389/fimmu.2019.03022 (PMC6996488; doi:10.3389/fimmu.2019.03022)
Supplement: Supplementary file 1 [file Data_Sheet_1.pdf]

Identification of novel genetic variants in CVID patients with autoimmunity, autoinflammation, or malignancy

Mette Christiansen1*, Rasmus Offersen2*, Jens Magnus Bernth Jensen1, Mikkel Steen Petersen1, Carsten S. Larsen2, and Trine H. Mogensen2,3,4

1) Department of Clinical immunology, Aarhus University Hospital (AUH)

2) Department of Infectious Diseases, AUH

3) Department of Biomedicine, Aarhus University (AU)

4) Department of Clinical Medicine, AU

Running title: Whole exome sequencing of CVID patients

Key words: common variable immunodeficiency (CVID), WES, autoimmunity, granuloma, malignancy, B cell phenotype

*these authors contributed equally

Corresponding author: Trine H. Mogensen, Department of Infectious Diseases, Palle Juul-Jensens Boulevard 99, 8200 Aarhus N, Denmark

Supplementary Tables and figures

ACP5, ACTB, ADA, ADA2, ADAM17, ADAMTS13, ADAR, ADRB2, AGA, AICDA, AIM2, AIRE, AK2, ALG13, ALOX5, AP1S3, AP3B1, APOL1, ARHGEF6, ARID5B, ARMS2, ATM, ATR, BACH2, BANK1, BCL10, BLM, BLNK, BLOC1S3, BLOC1S6, BRCA2, BRIP1, BTK, C1QA, C1QB, C1QC, C1R, C1S, C2, C3, C3AR1, C4A, C4B, C4BPA, C4BPB, C5, C5AR1, C5AR2, C6, C7, C8A, C8B, C8G, C9, CA2, CAPG, CARD11, CARD14, CARD9, CARMIL2, CASP1, CASP10, CASP8, CAVIN1, CAVIN1, CCDC103, CCDC114, CCL11, CD19, CD244, CD247, CD27, CD36, CD3D, CD3E, CD3G, CD4, CD40, CD40LG, CD46, CD55, CD59, CD70, CD79A, CD79B, CD81, CD8A, CDCA7, CDKN2B, CEBPE, CFB, CFD, CFH, CFHR1, CFHR2, CFHR3, CFHR4, CFHR5, CFI, CFP, CHD7, CHUK, CIITA, CLCN7, CLEC4D, CLEC6A, CLEC7A, CLPB, CNBP, COG6, COL7A1, COLEC10, COLEC11, COLEC12, COPA, CORO1A, CR1, CR2, CREBBP, CSF1R, CSF2RA, CSF3R, CTC1, CTLA4, CTPS1, CTSC, CXCL10, CXCR4, CYBA, CYBB, DCLRE1C, DDX11, DDX41, DDX58, DGKE, DHFR, DKC1, DNAI1, DNAJC21, DNAL1, DNASE1, DNASE1L3, DNMT3B, DNAAF5, DOCK2, DOCK8, DPP10, DTNBP1, ELANE, ELF4, EPG5, ERAP1, ERCC2, ERCC3, ERCC4, ERCC6L2, ETV6, F11, F12, F13A1, F13B, F5, F7, F8, F9, FADD, FANCA, FANCB, FANCC, FANCD2, FANCE, FANCF, FANCG, FANCI, FANCL, FANCM, FAS, FASLG, FCER1G, FCGR1A, FCGR2A, FCGR2B, FCGR3A, FCGR3B, FCGRT, FCN1, FCN2, FCN3, FERMT3, FGA, FGB, FLG, FOXN1, FOXP3, FPR1, FXII, G3BP1, G6PC, G6PC3, G6PD, GATA1, GATA2, GFI1, GINS1, GJC2, GP1BA, GP1BB, GP9, GPI, GTF2H5, HAX1, HELLS, HLA-C, HLA-DRB1, HNMT, HPS1, HPS3, HPS4, HPS5, HPS6, HTR1A, HTRA1, HYDIN, IBTK, ICOS, IFI16, IFIH1, IFITM3, IFNG, IFNGR1, IFNGR2, IGHG2, IGHM, IGHMBP1, IGKC, IGLL1, IKBKB, IKBKE, IKBKG, IKZF1, IKZF3, IL10, IL10RA, IL10RB, IL12B, IL12RB1, IL13, IL16, IL17F, IL17RA, IL17RB, IL17RC, IL18, IL1RN, IL2, IL20RA, IL21, IL21R, IL23A, IL2RA, IL2RG, IL36RN, IL7R, INO80, INSR, IRAK4, IRF3, IRF5, IRF7, IRF8, ISG15, ITCH, ITGAM, ITGB2, ITK, ITPKC, JAGN1, JAK2, JAK3, KMT2D, KRAS, LAMTOR2, LCK, LIG1, LIG4, LPIN2, LRBA, LRRC32, LRRC6, LRRC8A, LTA, LTBP3, LTBR, LYST, MAGT1, MAL, MALT1, MAN2B1, MANBA, MAP3K14, MAP3K7, MAPK8, MASP1, MASP2, MAVS, MB21D1, MBL2, MC2R, MCM3AP, MCM4, MCM9, MEFV, MKL1, MLPH, MMACHC, MOGS, MPL, MPO, MRE11, MRE11A, MS4A1, MS4A2, MSH5, MSH6, MSN, MST1, MTHFD1, MVK, MYD88, MYH9, MYO5A, MYO5B, MYSM1, NBN, NCF1, NCF2, NCF4, NCSTN, NFAT5, NFKB1, NFKB2, NFKBIA, NFKBIL1, NHEJ1, NHP2, NKX2-5, NLRC3, NLRC4, NLRP1, NLRP12, NLRP13, NLRP2, NLRP3, NLRP4, NLRP7, NME8, NOD2, NOP10, NRAS, NSMCE3, ORAI1, OSTM1, PADI4, PALB2, PARN, PBX1, PCCA, PCCB, PEPD, PGM3, PHF11, PI4KA, PIGA, PIK3CD, PIK3R1, PLA2G7, PLCG2, PLG, PMM2, PMS2, PNP, POLA1, POLE, PRF1, PRKCD, PRKD1, PRKDC, PROC, PROS1, PRPS1, PSENEN, PSMA2, PSMB8, PSTPIP1, PTCRA, PTEN, PTPN11, PTPN22, PTPRC, PTX3, PYCARD, RAB27A, RAC2, RAD50, RAD51C, RAG1, RAG2, RASGRP1, RASGRP2, RASGRP3, RBCK1, RBM8A, RECQL4, RET, RFX5, RFXANK, RFXAP, RHOH, RIPK1, RIPK3, RMRP, RNASEH2A, RNASEH2B, RNASEH2C, RNF168, RNF31, RORA, RORC, RPGR, RPL11, RPL15, RPL26, RPL35A, RPL36, RPL5, RPS10, RPS15, RPS15A, RPS17, RPS19, RPS24, RPS26, RPS27A, RPS28, RPS29, RPS7, RPSA, RSPH9, RTEL1, RUNX1, SAMHD1, SBDS, SCGB1A1, SCGB3A2, SCIMP, SEMA3E, SERAC1, SERPING1, SF3B6, SFTPB, SH2D1A, SH3BP2, SH3BP5, SIAE, SKIV2L, SLC15A4, SLC22A4, SLC29A3, SLC35A1, SLC35C1, SLC37A4, SLC39A4, SLC46A1, SLC7A7, SLX4, SMARCAL1, SOCS4, SP110, SPINK5, SRP72, STAT1, STAT2, STAT3, STAT4, STAT5A, STAT5B, STIM1, STK4, STX11, STXBP2, TANK, TAP1, TAP2, TAPBP, TAZ, TBC1D1, TBCE, TBK1, TBX1, TCF3, TCIRG1, TCN2, TERC, TERT, TFRC, TGFB1, THBD, TICAM1, TINF2, TIRAP, TLR2, TLR3, TLR4, TLR5, TLR7,

TLR8, TLR9, TMC6, TMC8, TMEM173, TNF, TNFAIP3, TNFRSF11A, TNFRSF13B, TNFRSF13C, TNFRSF1A, TNFRSF4, TNFSF12, TNFSF13B, TPP2, TRAC, TRADD, TRAF2, TRAF3, TRAF3IP2, TRAF6, TREM2, TREX1, TRNT1, TTC37, TTC7A, TYK2, UNC119, UNC13D, UNC93B1, UNG, UPB1, USB1, VAV1, VPREB1, VPS13B, VPS45, WAS, WDR1, WDR5, WIPF1, WRAP53, XIAP, XK, XRCC6, ZAP70, ZBTB24, ZNF296, ZNF341

Supplementary Table S1. Gene list

Case Samples	Gene Symbol	Transcript ID	Transcript Variant	Protein Variant	gnomAD Frequency	SIFT Function Prediction	PolyPhen-2 Function Prediction	CADD Score	MSC
**P2**	ERAP1	NM_016442.4	c.1432delC	p.L478fs*14	0			33	3.313
	ZNF319	NM_020807.2	c.272C>T	p.P91L	0.004	A	B	21.8	3.313
	JAK3	NM_000215.3	c.361C>T	p.R121C	0.001	D	PoD	23.3	0.001
**P3**	TREX1	NM_033629.5	c.923C>G	p.S308C	0.005	D	PoD	14.6	5.605
	DNASE1L3	NM_004944.3	c.97G>C	p.E33Q		T	PoD	21.3	16.330
**P4**	COL7A1	NM_000094.3	c.2528G>T	p.R843L	0	D	PoD	24.6	0.036
	TCF3	NM_003200.4	c.661C>G	p.L221V		T	PrD	23.2	3.313
	FANCB*	NM_001018113.2	c.1140T>A	p.F380L	0.026	T	B	13.12	0.001
**P5**	PCCB	NM_000532.4	c.62T>C	p.L21P		T	B	22.4	0.057
	DOCK8	NM_203447.4	c.2438C>T	p.T813I	0.004	T	B	22.5	0.001
**P7**	NLRP2	NM_017852.4	c.886G>A	p.A296T	0.001	D	B	12.1	3.313
**P8**	SMARCAL1	NM_014140.3	c.1762G>C	p.A588P	0	T	B	22.7	6.656
	INSR	NM_000208.3	c.3760T>C	p.Y1254H		D	B	18.2	0.069
	IL10RA	NM_001558.3	c.223T>C	p.C75R		D	PrD	23.3	0.034
	C5AR2	NM_018485.2	c.520C>T	p.R174W	0	D	PrD	23.7	14.180
**P9**	CFHR4	NM_001201550.3	c.579T>G	p.C193W		D	PrD	22.4	3.313
	PTPRC	NM_002838.4	c.3464A>C	p.E1155A		D	PrD	24.4	10.400
**P10**	IL2RG	NM_000206.2	c.-83G>A					21.9	0.001
**P11**	ERCC3	NM_000122.1	c.1544C>G	p.S515C		D	PoD	27.9	0.001
	TCN2	NM_000355.3	c.529G>A	p.D177N	0.001	T	B	16.3	0.001
**P13**	C3	NM_000064.3	c.125T>C	p.M42T	0	D	B	18.1	0.001
**P14**	SLC7A7	NM_001126106.2	c.914T>C	p.F305S	0.003	D	PrD	29.3	0.002
	MAVS	NM_020746.4	c.254C>T	p.A85V	0.004	T	PrD	24.5	3.313
**P15**	G6PC	NM_000151.3	c.230+1G>C					34	6.870
**P17**	EPG5	NM_020964.3	c.3895C>G	p.L1299V			B	16.5	0.006
**P18**	DOCK8	NM_203447.3	c.1943C>T	p.T648I		D	PrD	29	0.001
	TLR4	NM_138554.4	c.1144A>G	p.R382G	0	T	B	13.6	3.313
	TMC6	NM_007267.7	c.536G>A	p.R179Q	0.001	T	PrD	27.9	22.400
**P19**	RFXANK	NM_003721.3	c.421C>T	p.R141C	0.006	D	B	23.3	21.600

Supplementary Table S2
Additional variants identified, all variants are heterozygous except one homozygous variant found in P4 in the *FANCB* gene (*). A, Activating; D, damaging; T, Tolerated; B, Benign; PoD, Possibly Damaging; PrD, Probably Damaging.

Patient ID	Pneumococcal antibodies (prior IVIG; sufficient levels x/12 serotypes)	Pneumococcal immunization test (sufficient levels x/12 serotypes)	Predispositions	FEV1 % VC	Faecal calprotectin (mg/kg; max if several)	Microbiology	Referred from dept.	Prophylactic AB	IVIG
1	Performed prior IVIG, data not available	NP	none	NP	NP	Enterovirus (Faeces) x1	Rheumatology / Respiratory med.	Moxifloxacin	HyQvia
2	Performed prior IVIG, data not available	Immunized after IVIG substitution	Father: hyper-eosinophilic syndrome	NP	240	none pos.	Gastroenterology	none	Kiovig
3	After IVIG	NP	Sister: coeliac or Mb. crohn	80,9	117	none pos.	GP	none	Gamma-norm
4	NP	NP	none	83	NP	Enterobacteriaceae	Pulmonary medicine	none	Hizentra
5	After IVIG	NP	none	74	NP	*Streptococcus pneumoniae* in sputum. Oral candidiasis	Internal med.	Azitromycin	Hizentra
6	After IVIG	NP	none	104	NP	*Pseudomonas aeruginosa* (ascites)	unknown	Azitromycin	HyQvia
7	NP	NP	Grandmother: psoriasis. Grandfather and father: RA. Son IgG subclass deficiency.	99	NP	none pos.	unknown	Azitromycin	Hizentra
8	Normal	NP	Brother Mb. Crohn	NP	328	none pos.	Gastroenterology	Valacyclovir, Anakinra	Gamma-norm
9	NP	NP	none	NP	NP	*H. Influenzae* x 9 sputum + x 2	Haematology	Azitromycin	Privigen

10	NP	NP	mother: asthma, thyroiditis. Father: DMII	NP	1266	Sputum: *Haemophilus influenzae*, *Moxarella catarrhalis*, VZV, GBS	unknown	Sulfotrim	Hizentra
11	NP	NP	none	NP	NP	*Clostridiun difficile* (faeces)	Internal medicine	Unknown	Unknown
12	NP	NP	Father: pancreatic cancer (died 62), half-brother colon cancer (66)	NP	192	none pos.	Haematology	Sulfotrim	Gamma-norm
13	NP	NP	father and brother: cardiomyopathy	NP	392	none pos.	Gastroenterology	Azitromycin	HyQvia
14	NP	NP	none	82	1800	none pos.	unknown	none	Hizentra
15	Decreased	[7/12]	parents: irritable bowel diseases + thrombotic events	93	1147	*Clostridium difficile* (faeces)	Gastroenterology	none	none (anaphylactic reaction on IVIG)
16	Decreased	[5/12]	Mother + uncle colon cancer.	61	NP		Haematology	Azitromycin	HyQvia
17	Performed prior IVIG, data not available		none	91	NP		Haematology	Azitromycin	Gamma-norm
18	NP	NP	none	108	NP	none pos.	Haematology	Azitromycin	Hizentra
19	Performed prior IVIG, data not available	NP	none	NP	NP	none pos.	Pulmonary medicine	none	Hizentra
20	Decreased (6/12)	NP	Mother, brother: Sarcoidosis.	NP	NP	*Staph. aureus* (skin, tonsils)	Pulmonary medicine	Azitromycin	None

Supplementary Table S3. Additional clinical and paraclinical information.

patient ID	Naïve B cells CD19+IgD+CD27-CD38-	Transitional B cells CD19+IgD+CD27-CD38+	Class-switched memory CD19+IgD-CD27+CD38-	Plasmablasts CD19+IgD-CD27+CD38-	MZ-like CD19+IgD+CD27+CD38-	CD21 low CD19+CD21lowCD38-
1	92,8	0.9	0.2	0	5.1	11.6
2	#	#	#	#	#	#
3	53	7	1	0.1	30	11
4	19,4	13.3	0.3	0.5	63.8	73.3
5	77.8	9.3	3.7	0	0.9	24.1
6	73.2	22.7	0.3	0.2	0.7	14.7
7	29.2	65.1	0.1	0	0.3	0.8
8	67,9	16.4	1	0.4	6,5	7
9	71	23.4	0.6	0	1.2	7.3
10	65.6	32.2	0.6	0.1	0.4	4.3
11	75.3	11.2	1.4	0.4	7,2	14.6
12	75.2	20.9	0.1	0.3	2.8	5
13	80,9	5.7	0.5	0.2	1.3	34.9
14	75.3	4.5	0.1	0.1	19	36
15	44.1	3.6	23,6	0.2	20.3	8
16	39.3	1.7	2.1	1	3.5	9
17	61.3	19.3	0.6	0.2	12.4	34.1
18	79.7	12.4	0.1	0	5	24.1
19	87.3	0.6	1	0	7.7	21.6
20	81.4	2.1	2.6	0,9	10.7	4,2

Supplementary Table S4. B cell subsets. Central 95% prediction intervals for more than 140 blood donors in percentage (reference values): Naïve B cells (41-79);Transitional B cells (1.5-10); class-switched, memory (4.8-28); Plasmablasts (0.1-0.9), MZ-like (4.7-39); CD21 low (2.4-15). # No data available. Red: decreased values; Blue: increased values.

patient ID	Naive, % of CD4+ CD3+CD4+CD8-CCR7+CD27+CD45RA+	Central memory % of CD4+ CD3+CD4+CD8-CCR7+CD27+CD45RA+	Effector memory % of CD4+ CD3+CD4+CD8-CCR7-	Naive, % of CD8+ CD3+CD4-CD8+CCR7+CD27+CD45RA+	Central memory % of CD8+ CD3+CD4-CD8+CCR7+CD27+CD45RA-	Effector memory % of CD8+ CD3+CD4-CD8+CCR7-
1	7,6	47.0	41.0	3.7	22.4	72.8
2	#	#	#	#	#	#
3	14.4	57.0	27.5	23.5	18.8	56.8
4	8.8	75.0	12.3	8.0	9.6	81.9
5	7.1	37.4	53.1	3.2	4.2	92.2
6	2.3	36.2	59.5	2.5	2.9	93.2
7	6.4	56.4	35.4	4.4	3.5	91.6
8	3.2	4.0	91.9	2.8	0.5	96.0
9	0.6	23.1	73.1	13.7	23.3	3.1
10	7.5	58.2	30.8	11.6	18.5	66.6
11	22.3	52.7	21.1	6.4	1.2	91.3
12	23.3	62.5	10.9	16.9	13.1	66.6
13	21.5	63.5	11.8	39.4	28.6	29.8
14	57.3	24.9	16.7	13.7	3.1	82.8
15	47.5	45.5	5.3	65.0	17.2	17.3
16	62.4	25.0	11.5	44.5	7.1	47.9
17	4.4	59.9	32.9	12.6	10.8	76.0
18	20.8	64.6	12.3	31,7	5.8	61.6
19	24.1	55.6	18.6	44.9	13.8	41.0
20	79.8	0.1	7.1	33.0	0.0	67.4

No data available

Supplementary Table S5. T cell subsets. Numbers represent naïve, central memory and effector memory fractions of total CD4+ or CD8+ T cells respectively Reference values: Naive, % of CD4+ (25-73); Central memory, % of CD4+ (19-53); Effector memory, % of CD4+ (4.7-46); Naive, % of CD8+ (6.8-73); Central memory, % of CD8+ (2.4-26); Effector memory, % of CD8+ (18.3-90.2). Red: decreased values; Blue: increased values
